# Magnesium intake and all-cause mortality after stroke: a cohort study

**DOI:** 10.1186/s12937-023-00886-1

**Published:** 2023-10-30

**Authors:** Mengyan Wang, Jianhong Peng, Caili Yang, Wenyuan Zhang, Zicheng Cheng, Haibin Zheng

**Affiliations:** 1 Department of Neurology, The First People’s Hospital of Linhai, Taizhou, 317000 Zhejiang Province China; 2 Department of Neurology, Affiliated Yueqing Hospital, Wenzhou Medical University, Wenzhou, 325000 Zhejiang Province China; 3 Department of Neurology, Affiliated Jinhua Hospital, Zhejiang University School of Medicine, Jinhua, 321000 Zhejiang Province China

**Keywords:** All-cause mortality, Dietary, Dietary supplement, Magnesium, Stroke

## Abstract

**Background:**

Population-based studies have shown that adequate magnesium intake is associated with a lower risk of stroke and all-cause mortality. Whether adequate magnesium intake is important for reducing all-cause mortality risk after stroke remains unclear.

**Methods:**

We analyzed data from 917 patients with a self-reported history of stroke from the National Health and Nutrition Examination Survey (NHANES) 2007–2018. The total magnesium intake was calculated by summing the magnesium intake from dietary and dietary supplements, and then adjusting for total energy intake according to the nutrient density method. Mortality status was determined using public-use linked mortality files from 2019. Cox regression model and restricted cubic splines were used to explore the relationship between magnesium intake and all-cause mortality.

**Results:**

The average total magnesium intake across all patients was 251.0 (184.5–336.5) mg/d, and 321 (70.2%) males and 339 (73.7%) females had insufficient magnesium intake. During a median follow-up period of 5.3 years, 277 deaths occurred. After fully adjusting for confounding factors, total magnesium intake levels were inversely associated with all-cause mortality risk (HR per 1-mg/(100 kcal*d) increase, 0.97; 95% CI, 0.94–1.00; *p* = 0.017). Participants with the highest quartile of total magnesium intake (≥ 18.5 mg/(100 kcal*d)) had a 40% reduction in all-cause mortality risk compared to those with the lowest quartile (≤ 12.0 mg/(100 kcal*d)) (HR, 0.60; 95% CI, 0.38–0.94; *p* = 0.024). Stratified analyses showed that this inverse association was statistically significant in those who were older, female, without hypertension, and had smoking, normal renal function, and adequate energy intake. Dietary magnesium intake alone might be not related to all-cause mortality.

**Conclusions:**

Stroke survivors who consumed adequate amounts of magnesium from diet and supplements had a lower risk of all-cause mortality.

**Supplementary Information:**

The online version contains supplementary material available at 10.1186/s12937-023-00886-1.

## Introduction

Magnesium is an important metal element in the human body, and its content is behind only those of calcium, sodium, and potassium. It plays a key role in regulating many physiological functions, including glucose metabolism, protein synthesis, nucleic acid production, and muscle and nerve function [1]. However, a considerable portion of the US population (approximately 50%) has insufficient magnesium intake [2]. The European Food Safety Authority (EFSA) recommends a daily magnesium intake of 350 mg for men and 300 mg for women [3].

Magnesium deficiency is associated with an increased risk of a wide range of diseases, including hypertension [4], diabetes [5], metabolic syndrome [6], coronary heart disease [7], and stroke [5]. Magnesium deficiency is also associated with an increased risk of mortality. In a meta-analysis of 19 prospective cohort studies by Bagheri et al., each 100 mg/day increase in dietary magnesium intake was associated with a 6% and 5% reduced risk of all-cause and cancer mortality, respectively [8]. However, the participants in the included studies were usually from the general population, and the association between magnesium intake and mortality in a stroke cohort has not yet been evaluated.

We hypothesized that magnesium intake would be inversely associated with the risk of all-cause mortality in patients with stroke. Therefore, the aim of the present study was to assess the relationship between magnesium intake and all-cause mortality in a nationally representative sample of adults with stroke in the United States (US).

## Method

### Patient selection

We included data from the National Health and Nutrition Examination Survey (NHANES) survey cycles 2007–2008 to 2017–2018. The NHANES is a complex, multistage, probability sampling survey designed to assess the health and nutritional status of the non-institutionalized US population. The survey is conducted in two-year cycles by the Centers for Disease Control and Prevention (CDC) using in-person interviews, physical examinations, and laboratory tests. The study protocols were approved by the National Center for Health Statistics Ethics Review Board, and all participants signed the informed consent.

We identified stroke survivors aged ≥ 20 years based on their self-reported health conditions and medical history. Stroke survivors were those who answered yes to the question, “Has a doctor or other health professional ever told you that you had a stroke?” The question was asked, in the home, by trained interviewers using the Computer-Assisted Personal Interview (CAPI) system, which is programmed with built-in consistency checks to reduce data entry errors and also uses online help screens to assist interviewers in defining key terms used in the questionnaire. When unusual, inconsistent, or unrealistic responses were recorded, the interviewer was alerted immediately and instructed to verify or edit the initial response. Participants with missing data regarding magnesium intakes, mortality, or key covariates were excluded.

**Fig. 1:**
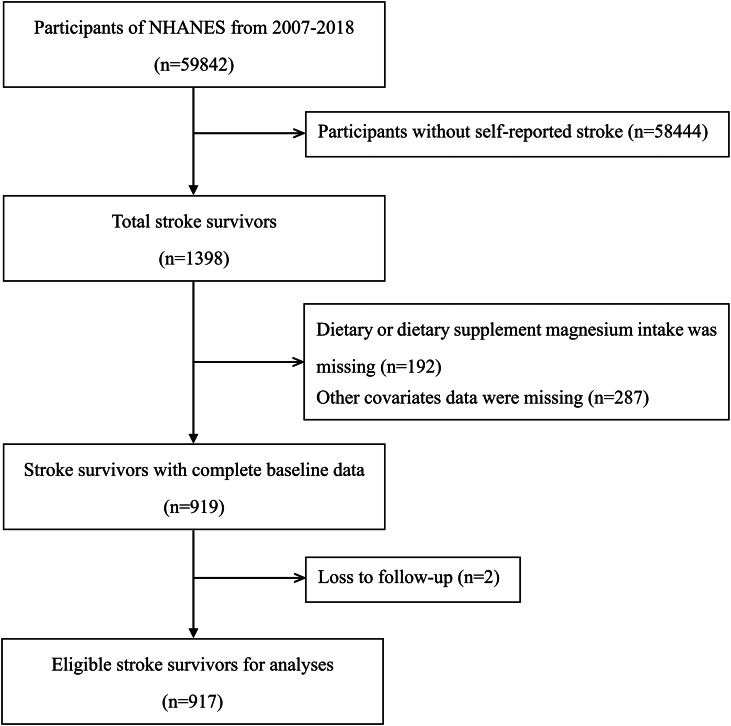
Flow chart of patient selection in this study

The process of study subject selection is shown in Fig. 1. A total of 1398 stroke survivors were identified in six cycles of NHANES, 2007–2008 to 2017–2018. Then, we excluded 192 participants with missing dietary or dietary supplement magnesium intake data, 287 participants with missing covariates data, and two participants with missing follow-up data. Finally, 917 stroke survivors were included in this study to analyze magnesium intake and mortality.

### Data collection

Information on dietary magnesium, other nutrients, and energy intakes were obtained from 24-hour dietary recall interviews. Participants were interviewed to the first dietary recall in-person in the mobile examination center (MEC), and the second interview was collected by telephone 3 to 10 days later. The types and amounts of foods, beverages, and water consumed during the 24-hour period prior to the interview were inquired to derive the dietary intake data. Dietary intakes of magnesium, sodium, potassium, calcium, fiber, and polyunsaturated fatty acids (PUFA) were averaged over the two recall periods. When only the first recall was available, this value was used instead of the average value. Furthermore, information on dietary supplement intakes were assessed by personal interview. The types and amounts of dietary supplements and antacids consumed during the 30-day period prior to the interview were inquired to derive a mean daily intake data. The total magnesium intake was calculated by summing the magnesium intake from diet and supplements, and then adjusting for total energy intake according to the nutrient density method [9].

Sociodemographic characteristics, including age, sex, race, education level, and ratio of family income to poverty (PIR) were obtained from self-reports. Body mass index (BMI) was calculated as weight in kilograms divided by height in meters squared. Vascular risk factors, including hypertension, diabetes, hyperlipidemia, coronary heart disease, congestive heart failure, smoking history, and drinking history were determined by self-reports and medical examination data. Hypertension was defined as self-reported high blood pressure, systolic blood pressure ≥ 140 mmHg, or diastolic blood pressure ≥ 90 mmHg. Diabetes was defined as self-reported diagnosis, fasting blood glucose ≥ 7.0 mmol/L, or hemoglobin A1c ≥ 6.5%. Hyperlipidemia was defined as self-reported high cholesterol level or total cholesterol ≥ 5.17 mmol/L. Coronary heart disease and congestive heart failure were defined as self-reported diagnosis. Smoking history was defined as smoking at least 100 cigarettes ever. Drinking history was defined as the consumption of at least 12 drinks of any one year.

### Mortality

The primary outcome measure was all-cause mortality. Mortality status was determined by using a probabilistic match between NHANES and the National Death Index (NDI) death certificate records [10]. The primary identifiers used in the linkages were: Social Security Number, first name, middle initial, last name or father’s surname, month of birth, day of birth, year of birth, state of birth, state of residence, race, and sex. A probability cutoff of 0.99 was selected for matches to have the lowest linkage errors (2%) possible. The follow-up time was determined as the period from the first dietary recall interviews date to the date of death or end of follow-up (December 31, 2019).

### Statistical analysis

The weighted median and interquartile range were used to present continuous variables, and the unweighted frequency and unweighted percentage were used to present categorical variables. The Wilcoxon rank-sum test for complex survey samples and the chi-squared test with Rao and Scott’s second-order correction were used to compare intergroup differences in continuous and categorical variables, respectively. The total magnesium intake was divided into quartile groups (Q1, Q2, Q3, and Q4). Kaplan–Meier curves were generated and compared among the total magnesium intake quartile groups.

Cox regression analysis was performed to examine the association between total magnesium intake and all-cause mortality. Four multivariable Cox regression models were evaluated: Model 1 was adjusted for age, sex, and race; Model 2 was adjusted for sociodemographic characteristics and lifestyles; Model 3 was adjusted for all variables in Model 2 plus dietary factors; Model 4 was adjusted for all variables in Model 3 and past medical history. A restricted cubic splines regression model with four knots (5th, 35th, 65th, and 95th percentiles) was used to examine the possibility of the non-linear association between total magnesium intake and all-cause mortality. Stratified analyses according to age, sex, and other variables were conducted and adjusted for the variables included in Model 4. Interaction analysis between total magnesium intake and each stratified variable was also tested. All statistical tests were two-tailed, and *p* < 0.05 was considered statistically significant. Statistical analyses were performed using the R version 4.2.1 (R Foundation for Statistical Computing, Vienna, Austria).

## Results

There were no notable differences in most aspects between all NHANES stroke survivors and those with complete information needed to be included in this analysis; the exceptions were that those included in the analysis were younger and had longer follow-up time and lower proportion of death (Supplementary Table 1).

### Patient characteristics

The average age of the stroke patients was 67.0 years, and 50.2% were women, of whom 8.1% were Mexican American, 6.1% other Hispanic, 51.7% White, 27.3% Black, and 6.9% other races and ethnicities. There were 277 deaths (30.2%) in this stroke survivor cohort over a median follow-up time of 5.3 (2.8–8.6) years. The average total magnesium intake across all patients was 251.0 (184.5–336.5) mg/d. According to EFSA recommended standard, 321 (70.2%) males and 339 (73.7%) females had insufficient magnesium intake. After considering energy intake, the average total magnesium intake was 14.5 (12.0–18.4) mg/(100 kcal*d). The quartiles of total magnesium intake levels were Q1 (≤ 12.0 mg/(100 kcal*d)), Q2 (12.1–14.5 mg/(100 kcal*d)), Q3 (14.6–18.4 mg/(100 kcal*d)), and Q4 (≥ 18.5 mg/(100 kcal*d)). Compared with survivors, those who died during follow-up were older, had higher PIR, serum creatinine, and total potassium and calcium intakes levels, had a higher proportion of hypertension, congestive heart failure, and coronary heart disease, and had lower BMI and total energy intake levels (all *p* < 0.05, Table 1).

**Table 1:** Clinical characteristics of patients

	Overall (n = 917)	Alive (n = 640)	Dead (n = 277)	*P*
Age, years	66.0 (55.0–76.0)	63.0 (52.0–71.0)	78.0 (68.0–80.0)	< 0.001
Sex				0.63
Female	460 (50.2%)	339 (53.0%)	121 (43.7%)	
Male	457 (49.8%)	301 (47.0%)	156 (56.3%)	
Race				0.10
Mexican American	74 (8.1%)	57 (8.9%)	17 (6.1%)	
Other Hispanic	56 (6.1%)	46 (7.2%)	10 (3.6%)	
Non-Hispanic White	474 (51.7%)	292 (45.6%)	182 (65.7%)	
Non-Hispanic Black	250 (27.3%)	194 (30.3%)	56 (20.2%)	
Other Race	63 (6.9%)	51 (8.0%)	12 (4.3%)	
Education				0.073
High school or less	560 (61.1%)	373 (58.3%)	187 (67.5%)	
Some college	242 (26.4%)	180 (28.1%)	62 (22.4%)	
College graduate	115 (12.5%)	87 (13.6%)	28 (10.1%)	
PIR				< 0.001
< 1	226 (24.6%)	170 (26.6%)	56 (20.2%)	
1–2	334 (36.4%)	218 (34.1%)	116 (41.9%)	
2–3	148 (16.1%)	98 (15.3%)	50 (18.1%)	
≥ 3	209 (22.8%)	154 (24.1%)	55 (19.9%)	
BMI, kg/m^2^	29.2 (24.9–34.1)	29.9 (25.5–34.6)	27.5 (24.0–31.0)	< 0.001
Hypertension	734 (80.0%)	497 (77.7%)	237 (85.6%)	0.001
Diabetes	356 (38.8%)	237 (37.0%)	119 (43.0%)	0.30
Hyperlipidemia	651 (71.0%)	474 (74.1%)	177 (63.9%)	0.14
Congestive heart failure	155 (16.9%)	90 (14.1%)	65 (23.5%)	0.003
Coronary heart disease	161 (17.6%)	97 (15.2%)	64 (23.1%)	0.005
Smoking	575 (62.7%)	390 (60.9%)	185 (66.8%)	0.17
Drinking	560 (61.1%)	385 (60.2%)	175 (63.2%)	0.32
Serum creatinine, µmol/L	84.0 (69.0–102.5)	81.3 (67.2–94.6)	97.2 (76.8–119.3)	< 0.001
Total energy, kcal/d	1759.0 (1299.1–2192.0)	1790.3 (1327.2–2298.2)	1625.2 (1251.9–2063.4)	0.020
Total PUFA, g/(100 kcal*d)	0.8 (0.7–1.0)	0.8 (0.7–1.1)	0.8 (0.7–1.0)	0.66
Total fiber, g/(100 kcal*d)	0.7 (0.6–1.0)	0.7 (0.6–1.0)	0.8 (0.6–1.0)	0.054
Total sodium, mg/(100 kcal*d)	165.0 (140.3–193.9)	165.4 (142.6–193.6)	160.3 (135.4–194.4)	0.36
Total potassium, mg/(100 kcal*d)	133.8 (107.8–159.7)	128.9 (104.7–158.1)	142.2 (120.5–162.0)	0.017
Total calcium, mg/(100 kcal*d)	50.0 (35.6–72.9)	47.2 (33.9–69.0)	56.3 (40.0–80.2)	0.004
Total magnesium, mg/(100 kcal*d)	14.5 (12.0–18.0)	14.3 (11.8–18.0)	15.2 (12.5–18.1)	0.20
Quartiles of total magnesium				0.12
Q1: ≤ 12.0 mg/(100 kcal*d)	230 (25.1%)	172 (26.9%)	58 (20.9%)	
Q2: 12.1–14.5 mg/(100 kcal*d)	228 (24.9%)	160 (25.0%)	68 (24.5%)	
Q3: 14.6–18.4 mg/(100 kcal*d)	229 (25.0%)	144 (22.5%)	85 (30.7%)	
Q4: ≥ 18.5 mg/(100 kcal*d)	230 (25.1%)	164 (25.6%)	66 (23.8%)	

BMI, body mass index; PIR, ratio of family income to poverty; PUFA, polyunsaturated fatty acids

### Total magnesium intake and all-cause mortality

**Fig. 2:**
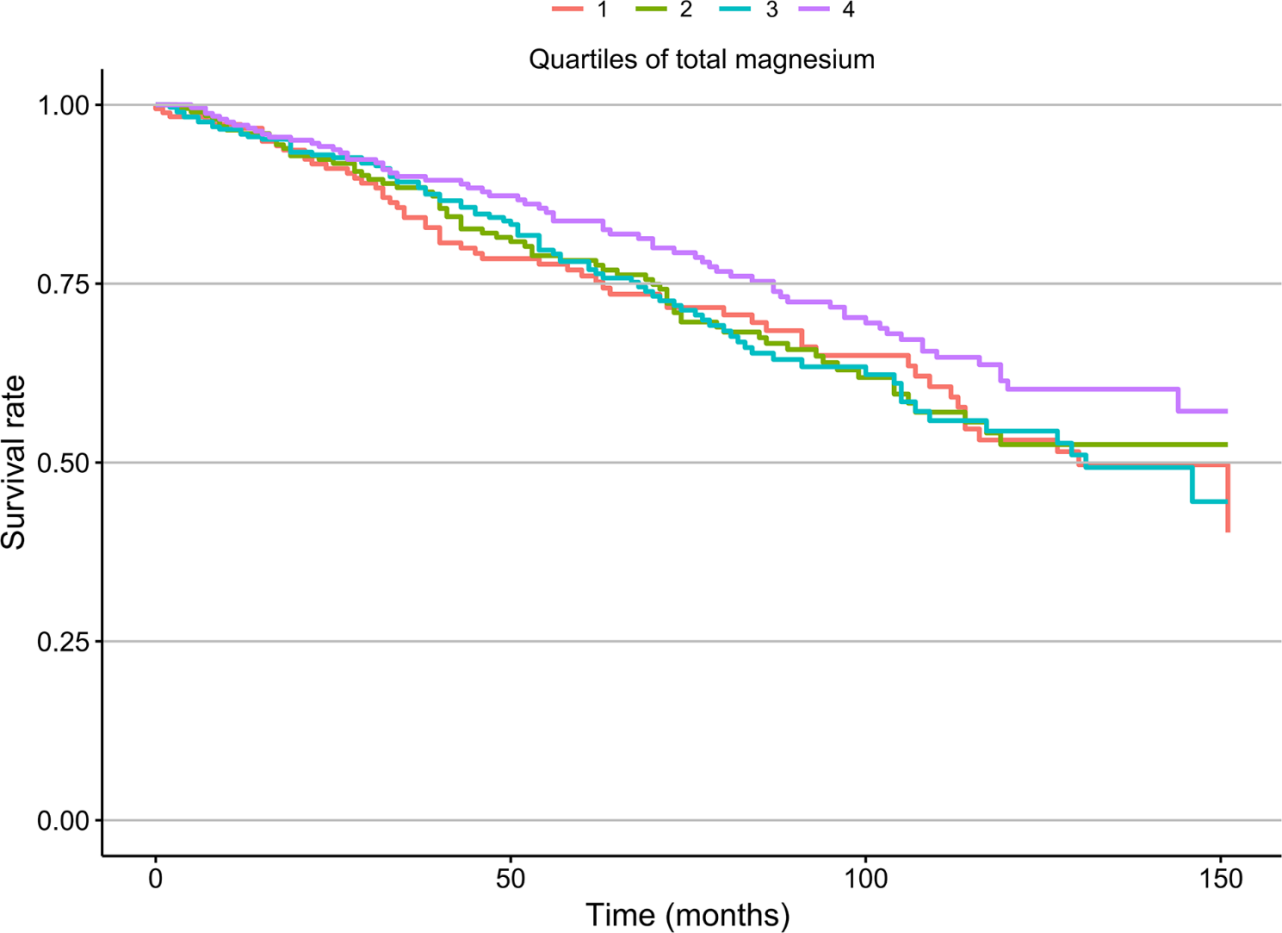
The Kaplan–Meier curve for the study participants with different total magnesium intake. The curve was adjusted for the same confounding variables as in Model 4

**Fig. 3:**
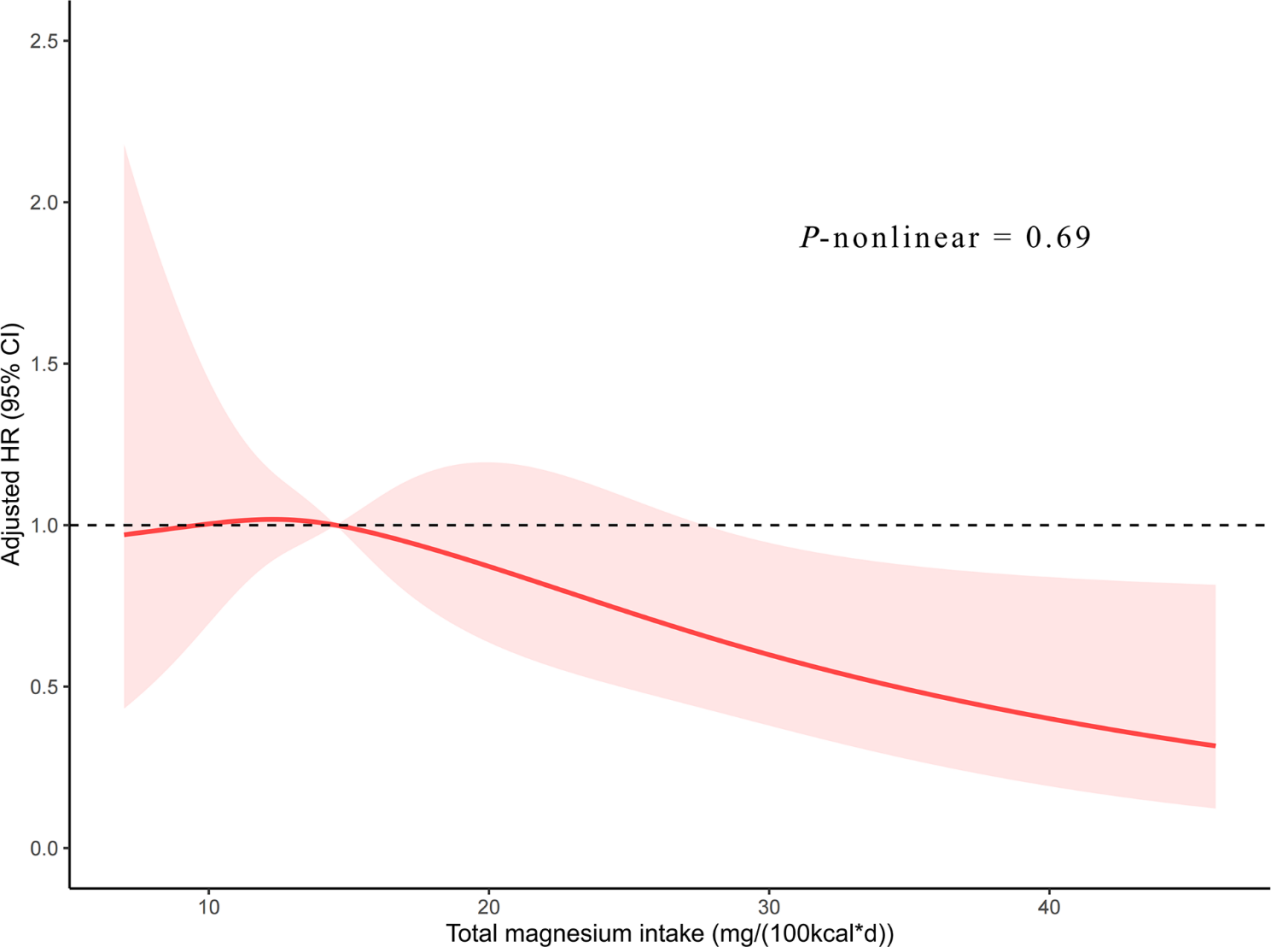
Restricted cubic spline regression model of the relationship between total magnesium intake and risk of all-cause mortality after stroke. The model was adjusted for the same confounding variables as in Model 4. CI, confidence interval; HR, hazard ratio

In the univariate analysis, there was no notable difference of total magnesium intake between patients who died and those who survived (15.2 vs. 14.3 mg/(100 kcal*d), *p* = 0.20, Table 1). However, in any multivariate model that adjusted for potential confounders, total magnesium intake levels had an independent inverse association with all-cause mortality risk. After fully adjusting for potential confounding factors, each 1-mg/(100 kcal*d) increase in total magnesium intake was associated with a 3% reduced risk of all-cause mortality (HR, 0.97; 95% CI, 0.94–1.00; *p* = 0.017; Table 2). Patients in the fourth quartile of total magnesium intake showed a statistically significant decline in all-cause mortality risk compared to those in the first quartile (HR, 0.60; 95% CI, 0.38–0.94; *p* = 0.024). In the multivariable-adjusted Kaplan–Meier curves, a substantially lower risk of death was observed among patients with the highest quartile of total magnesium intake (Fig. 2). The multivariable-adjusted spline regression model further confirmed that the inverse association between total magnesium intake and all-cause mortality risk was linear (*p*-nonlinear = 0.69, Fig. 3). Stratified analyses showed that the statistically significant association between total magnesium intake and all-cause mortality was observed in patients who were older, female, without hypertension, and had smoking, normal renal function, and adequate energy intake (Table 3). The multivariable Cox regression model showed that total sodium, potassium, and calcium intakes were not related to all-cause mortality risk (Supplementary Table 2).

**Table 2:** Cox regression analysis to identify the association between total magnesium intake and all-cause mortality

	Model 1		Model 2		Model 3		Model 4		
	HR (95% CI)	*P*	HR (95% CI)	*P*	HR (95% CI)	*P*	HR (95% CI)	*P*	
Total magnesium, per 1-mg/(100 kcal*d) increase	0.96 (0.94– 0.98)	< 0.001	0.96 (0.94– 0.98)	< 0.001	0.97 (0.94– 0.99)	0.009	0.97 (0.94– 1.00)	0.017	
Quartiles of total magnesium									
Q1: ≤ 12.0 mg/(100 kcal*d)	Ref		Ref		Ref		Ref		
Q2: 12.1–14.5 mg/(100 kcal*d)	0.93 (0.60– 1.45)	0.75	0.98 (0.62– 1.56)	0.95	1.02 (0.65– 1.59)	0.95	0.90 (0.58– 1.40)	0.65	
Q3: 14.6–18.4 mg/(100 kcal*d)	0.94 (0.65– 1.36)	0.74	0.91 (0.62– 1.34)	0.64	0.98 (0.65– 1.47)	0.91	0.92 (0.61– 1.39)	0.69	
Q4: ≥ 18.5 mg/(100 kcal*d)	0.56 (0.37– 0.84)	0.005	0.53 (0.34– 0.83)	0.005	0.60 (0.38– 0.95)	0.031	0.60 (0.38– 0.94)	0.024	
*P* for trend	0.006		0.003		0.031		0.037		

Model 1: adjusted for age, sex, and race

Model 2: adjusted for age, sex, race, education, PIR, BMI, smoking, drinking, and total energy

Model 3: adjusted for all variables in Model 2 plus total PUFA, total fiber, total sodium, total potassium, and total calcium

Model 4: adjusted for all variables in Model 3 plus hypertension, diabetes, hyperlipidemia, congestive heart failure, coronary heart disease, smoking, drinking, and serum creatinine

BMI, body mass index; CI, confidence interval; HR, hazard ratio; PIR, ratio of family income to poverty; PUFA, polyunsaturated fatty acids

**Table 3:** Stratified analyses to identify variables that may modify the association between total magnesium intake (per 1-mg/(100 kcal*d) increase) and all-cause mortality

	Number	HR (95% CI)	*P*	*P* for interaction
Age, years				
< 60	251	0.92 (0.73–1.18)	0.52	
≥ 60	666	0.97 (0.94–0.99)	0.012	0.52
Sex				
Female	460	0.96 (0.93–1.00)	0.031	
Male	457	0.98 (0.94–1.02)	0.25	0.44
BMI, kg/m^2^				
< 28	397	0.97 (0.93–1.01)	0.13	
≥ 28	520	0.99 (0.96–1.03)	0.70	0.007
Hypertension				
No	183	0.89 (0.82–0.97)	0.007	
Yes	734	0.99 (0.96–1.02)	0.37	0.074
Diabetes				
No	561	0.96 (0.92–1.00)	0.067	
Yes	356	0.99 (0.94–1.04)	0.64	0.29
Hyperlipidemia				
No	266	0.95 (0.89–1.00)	0.050	
Yes	651	0.98 (0.95–1.01)	0.24	0.96
Smoking				
No	342	0.97 (0.93–1.01)	0.14	
Yes	575	0.95 (0.91–1.00)	0.028	0.11
Serum creatinine, µmol/L				
< 100	620	0.96 (0.92–1.00)	0.032	
≥ 100	297	0.98 (0.94–1.02)	0.31	0.43
Energy, kcal/d				
< 2000	630	0.98 (0.96–1.01)	0.32	
≥ 2000	287	0.93 (0.89–0.97)	< 0.001	0.15

BMI, body mass index; CI, confidence interval; HR, hazard ratio

### Dietary magnesium intake and all-cause mortality

Among the included stroke patients, only approximately one-third of participants (276/917) had magnesium intake from dietary supplements. Thus, we additionally analyzed the relationship between dietary magnesium intake and all-cause mortality. The average dietary magnesium intake across all patients was 13.7 (11.5–16.8) mg/(100 kcal*d). Dietary magnesium intake was inversely associated with all-cause mortality risk in Models 1 and 2 (HR per 1-mg/(100 kcal*d) increase, 0.95; 95% CI, 0.91–0.99; *p* = 0.012; Supplementary Table 3). However, when dietary factors or past medical history was considered, the above association became insignificant (HR per 1-mg/(100 kcal*d) increase, 0.96; 95% CI, 0.91–1.01; *p* = 0.13).

### Discussion

In this cohort study of nationally representative sample of stroke survivors, a linear inverse association was found between total magnesium intake and risk of all-cause mortality. All-cause mortality decreased by 40% during follow-ups after a median of 5.3 years in the participants with the highest quartiles of total magnesium intake (≥ 18.5 mg/(100 kcal*d)). Dietary magnesium intake alone was not related to all-cause mortality. No statistically significant associations were observed between dietary sodium, potassium, or calcium intake and all-cause mortality.

Insufficient magnesium intake was found in approximately three quarters of stroke survivors, indicating that magnesium deficiency is quite common. Almost 64–67% of the US adults consumed less than the recommended standard of magnesium from food in 2001–2002, and the estimate decreased to 53–56% in 2005–2006 [11]. Magnesium intake level was 31% below the dietary reference intakes in the French adult in 2006–2007, and the figure reduced to 19% in 2014–2015 [12]. Although magnesium consumption in the population is gradually increasing, it is still far below the reference level, particularly in patients with stroke.

The results on the association between magnesium intake and all-cause mortality are in line with previous findings that focused on community populations or cancer patients. In a nationally representative sample of US adults, adequate intake of magnesium was associated with reduced all-cause mortality during a median follow-up of 6.1 years [13]. Another study conducted in the Mediterranean adults with high cardiovascular risk in Spain reported that participants in the highest tertile of dietary magnesium intake had a 37% reduction in all-cause mortality risk compared to those in the lowest tertile [14]. Two studies conducted in Chinese community populations showed decreased all-cause mortality in the individuals with a higher dietary magnesium intake; however, this association depended on dietary quality and the ratio of calcium to magnesium intake [15, 16]. An inverse association between total magnesium intake and all-cause mortality has also been observed in patients with breast and colorectal cancers [17, 18]. Our study provides new evidence for this inverse association in the stroke population and confirms that this association is linear.

It is plausible that the beneficial effect of high magnesium intake on all-cause mortality in stroke survivors could be due to neuroprotective role of magnesium. A drawback of our study was that the serum magnesium concentration was not measured. A multicenter study revealed that acute ischemic stroke patients with the lowest serum magnesium levels (< 0.82mmol/L) had two-fold increase in the risk of in-hospital mortality [19]. Magnesium levels in cerebrospinal fluid were also lower in patients with acute ischemic stroke who died within seven days than in those who survived [20]. Magnesium has several crucial roles in neuroprotection, including blockade of *N*-methyl D-aspartate (NMDA) receptor–mediated excitotoxic injury, calcium antagonism, vasodilatation, and antiplatelet effect [21]. The protective effect of magnesium was particularly evident in some subgroups of stroke patients, such as the elderly and those who smoked. Aging [22] and smoking [23] are associated with elevated levels of inflammation in the human body. Magnesium has anti-inflammatory effects and magnesium intake is inversely associated with serum C-reactive protein and interleukin-6 levels [24, 25]. Thus, ensuring sufficient magnesium intake is important for these subgroups. Total energy intake exceeding 2000 kcal per day ensures sufficient daily magnesium intake.

Total calcium intake independent of magnesium intake was not associated with all-cause mortality. Some studies have reported an association between calcium intake and all-cause mortality; however, most have not considered magnesium intake [26]. Calcium is essential for intracellular signalling, cardiac and vascular smooth muscle contraction, and coagulation pathway, but high levels may also contribute to atheromatous plaques [27].

Some limitations should be acknowledged in our study. First, the participants of this study were stroke survivors rather than acute stroke patients. The length of time from stroke onset to the first dietary recall interviews was unclear. Second, the diagnosis of stroke was determined by participants’ self-reports, which might introduce a false reporting. The survey questionnaire also did not distinguish between ischemic and hemorrhagic stroke. Third, serum magnesium concentration was not measured in this study. It is unclear whether the impact of total magnesium intake on all-cause mortality is mediated by serum magnesium levels. Fourth, some important confounding variables (e.g., stroke severity and adherence to secondary prevention of stroke) that might influence mortality were not available. Fifth, subgroup analysis was exploratory and the results need to be validated in an independent cohort.

## Conclusion

In conclusion, we found that magnesium intake is inversely associated with the risk of all-cause mortality in stroke survivors, especially total magnesium intake. Considering that insufficient magnesium intake is common in stroke patients, improving magnesium intake in stroke survivors is warranted. Further randomized control trials are needed to explore whether increasing dietary and dietary supplement magnesium intake can reduce the risk of death after stroke.

### Electronic supplementary material

Below is the link to the electronic supplementary material.

Supplementary Material 1

## Data Availability

The datasets generated and analysed during the current study are available from the corresponding author on reasonable request.

## Abbreviations

BMI: Body mass index
CAPI: Computer-Assisted Personal Interview
CDC: Centers for Disease Control and Prevention
CI: Confidence interval
EFSA: European Food Safety Authority
HR: Hazard ratio
MEC: Mobile examination center
NDI: National Death Index
NHANES: National Health and Nutrition Examination Survey
NMDA: *N*-methyl D-aspartate
PIR: Ratio of family income to poverty
PUFA: Polyunsaturated fatty acids
US: United States
