# Correction: Magnesium intake and all-cause mortality after stroke: a cohort study

**DOI:** 10.1186/s12937-023-00892-3

**Published:** 2023-11-14

**Authors:** Mengyan Wang, Jianhong Peng, Caili Yang, Wenyuan Zhang, Zicheng Cheng, Haibin Zheng

**Affiliations:** 1 Department of Neurology, The First People’s Hospital of Linhai, Taizhou, 317000 Zhejiang Province China; 2 Department of Neurology, Affiliated Yueqing Hospital, Wenzhou Medical University, Wenzhou, 325000 Zhejiang Province China; 3 Department of Neurology, Affiliated Jinhua Hospital, Zhejiang University School of Medicine, Jinhua, 321000 Zhejiang Province China

Nutrition Journal (2023) 22:54

10.1186/s12937-023-00886-1

Following the publicaiton of the original article [1], the author reported that In Fig. 1, the number of participants without self-reported stroke was 99,119 when it should be 58,444.

The original article has been corrected.
