# Supplementary material for: Magnesium intake and all-cause mortality after stroke: a cohort study
Source: Nutr J. 2023 Oct 30;22:54. doi: 10.1186/s12937-023-00886-1 (PMC10614364; doi:10.1186/s12937-023-00886-1)
Supplement: Supplementary file 1 — Supplementary Material 1 [file 12937_2023_886_MOESM1_ESM.docx]

**Magnesium intake and all-cause mortality after stroke: A cohort study**

**Table S1.** Characteristics of NHANES stroke survivors

**Table S2.** Cox regression analysis to identify potential risk factors of all-cause mortality after stroke

**Table S3.** Cox regression analysis to identify the association between dietary magnesium intake and all-cause mortality

Table S1. Characteristics of NHANES stroke survivors

|  | All NHANES stroke survivors (n = 1398) | NHANES stroke survivors included in this analysis (n = 917) | *P* |
| --- | --- | --- | --- |
| Age | 68.0 (59.0–78.0) | 67.0 (58.0–77.0) | 0.054 |
| Sex |  |  | 0.62 |
| Female | 716 (51.2%) | 460 (50.2%) |  |
| Male | 682 (48.8%) | 457 (49.8%) |  |
| Race |  |  | 0.54 |
| Mexican American | 123 (8.8%) | 74 (8.1%) |  |
| Other Hispanic | 93 (6.7%) | 56 (6.1%) |  |
| Non-Hispanic White | 674 (48.2%) | 474 (51.7%) |  |
| Non-Hispanic Black | 396 (28.3%) | 250 (27.3%) |  |
| Other Race | 112 (8.0%) | 63 (6.9%) |  |
| Education |  |  | 0.70 |
| High school or less | 866 (62.1%) | 560 (61.1%) |  |
| Some college | 347 (24.9%) | 242 (26.4%) |  |
| College graduate | 182 (13.0%) | 115 (12.5%) |  |
| PIR |  |  | 0.83 |
| <1 | 335 (26.4%) | 226 (24.6%) |  |
| 1–2 | 449 (35.3%) | 334 (36.4%) |  |
| 2–3 | 199 (15.7%) | 148 (16.1%) |  |
| ≥ 3 | 288 (22.7%) | 209 (22.8%) |  |
| BMI, kg/m^2^ | 28.9 (24.9–33.3) | 29.0 (25.1–33.5) | 0.70 |
| Hypertension | 1117 (79.9%) | 734 (80.0%) | 0.93 |
| Diabetes | 544 (38.9%) | 356 (38.8%) | 0.97 |
| Hyperlipidemia | 956 (69.0%) | 651 (71.0%) | 0.30 |
| Congestive heart failure | 250 (18.1%) | 155 (16.9%) | 0.47 |
| Coronary heart disease | 256 (18.6%) | 161 (17.6%) | 0.52 |
| Smoking | 834 (59.7%) | 575 (62.7%) | 0.14 |
| Drinking | 694 (59.3%) | 560 (61.1%) | 0.42 |
| Serum creatinine, μmol/L | 86.6 (70.7–107.9) | 87.5 (71.6–107.9) | 0.90 |
| Total energy, kcal/d | 1640.5 (1217.0–2107.8) | 1694.0 (1245.5–2141.0) | 0.24 |
| Total PUFA, g/(100kcal*d) | 0.8 (0.7–1.0) | 0.8 (0.7–1.0) | 0.69 |
| Total fiber, g/(100kcal*d) | 0.8 (0.6–1.0) | 0.8 (0.6–1.0) | 0.70 |
| Total sodium, mg/(100kcal*d) | 165.0 (139.8–194.7) | 164.7 (139.7–194.7) | 0.92 |
| Total potassium, mg/(100kcal*d) | 132.1 (107.6–161.2) | 132.0 (108.5–159.3) | 0.86 |
| Total calcium, mg/(100kcal*d) | 49.7 (34.8–71.2) | 49.0 (34.6–70.5) | 0.59 |
| Total magnesium, mg/(100kcal*d) | 14.8 (12.0–18.8) | 14.5 (12.0–18.4) | 0.49 |
| Death | 476 (34.1%) | 277 (30.2%) | 0.051 |
| Follow-up time, month | 59.0 (31.0–99.0) | 63.0 (34.0–103.0) | 0.023 |

BMI, body mass index; PIR, ratio of family income to poverty; PUFA, polyunsaturated fatty acids.

Table S2. Cox regression analysis to identify potential risk factors of all-cause mortality after stroke

|  | HR (95% CI) | *P* |
| --- | --- | --- |
| Age, years | 1.10 (1.08–1.12) | <0.001 |
| Sex |  |  |
| Female | Ref |  |
| Male | 0.78 (0.52–1.17) | 0.23 |
| Race |  |  |
| Mexican American | Ref |  |
| Other Hispanic | 0.46 (0.15–1.36) | 0.16 |
| Non-Hispanic White | 1.03 (0.53–2.03) | 0.92 |
| Non-Hispanic Black | 0.94 (0.45–1.97) | 0.88 |
| Other Race | 1.02 (0.46–2.27) | 0.96 |
| Education |  |  |
| High school or less | Ref |  |
| Some college | 0.79 (0.50–1.25) | 0.31 |
| College graduate | 0.98 (0.54–1.80) | 0.96 |
| PIR |  |  |
| < 1 | Ref |  |
| 1–2 | 1.01 (0.64–1.60) | 0.96 |
| 2–3 | 0.99 (0.54–1.82) | 0.98 |
| ≥ 3 | 0.58 (0.33–1.00) | 0.052 |
| BMI, kg/m^2^ | 0.96 (0.93–1.00) | 0.052 |
| Hypertension | 0.97 (0.64–1.46) | 0.89 |
| Diabetes | 1.18 (0.81–1.71) | 0.38 |
| Hyperlipidemia | 0.68 (0.51–0.90) | 0.008 |
| Congestive heart failure | 1.75 (1.21–2.53) | 0.003 |
| Coronary heart disease | 1.53 (1.09–2.14) | 0.013 |
| Smoking | 1.64 (1.19–2.25) | 0.002 |
| Drinking | 0.81 (0.58–1.13) | 0.22 |
| Serum creatinine, per 10-μmol/L increase | 1.04 (1.02–1.06) | <0.001 |
| Total energy, per 100-kcal/d increase | 1.00 (0.97–1.03) | 0.90 |
| Total PUFA, per 1-g/(100kcal*d) increase | 1.00 (0.59–1.71) | 0.99 |
| Total fiber, per 1-g/(100kcal*d) increase | 0.55 (0.35–0.86) | 0.008 |
| Total sodium, per 10-mg/(100kcal*d) increase | 0.99 (0.95–1.04) | 0.75 |
| Total potassium, per 10-mg/(100kcal*d) increase | 1.03 (0.99–1.08) | 0.15 |
| Total calcium, per 10-mg/(100kcal*d) increase | 1.00 (0.98–1.03) | 0.87 |
| Total magnesium, per 1-mg/(100kcal*d) increase | 0.97 (0.94–1.00) | 0.017 |

BMI, body mass index; CI, confidence interval; HR, hazard ratio; PIR, ratio of family income to poverty; PUFA, polyunsaturated fatty acids.

Table S3. Cox regression analysis to identify the association between dietary magnesium intake and all-cause mortality

|  | Model 1 | | Model 2 | | Model 3 | | Model 4 | |  |  |
| --- | --- | --- | --- | --- | --- | --- | --- | --- | --- | --- |
|  | HR (95% CI) | *P* | HR (95% CI) | *P* | HR (95% CI) | *P* | HR (95% CI) | *P* |  |  |
| Dietary magnesium, per 1-mg/(100kcal*d) increase | 0.95 (0.92– 0.98) | 0.002 | 0.95 (0.91– 0.99) | 0.012 | 0.95 (0.91– 1.00) | 0.060 | 0.96 (0.91– 1.01) | 0.13 |  |  |
| Quartiles of dietary magnesium |  |  |  |  |  |  |  |  |  |  |
| Q1: ≤ 11.5 mg/(100kcal*d) | Ref | | Ref | | Ref | | Ref | |  |  |
| Q2: 11.6–13.7 mg/(100kcal*d) | 0.71 (0.46– 1.08) | 0.11 | 0.62 (0.39– 0.99) | 0.044 | 0.62 (0.38– 1.03) | 0.067 | 0.64 (0.39– 1.05) | 0.078 |  |  |
| Q3: 13.8–16.8 mg/(100kcal*d) | 0.97 (0.69– 1.36) | 0.86 | 0.91 (0.68– 1.23) | 0.55 | 0.96 (0.61– 1.51) | 0.86 | 0.97 (0.60– 1.59) | 0.92 |  |  |
| Q4: ≥ 16.9 mg/(100kcal*d) | 0.50 (0.33– 0.75) | <0.001 | 0.48 (0.30– 0.76) | 0.002 | 0.52 (0.29– 0.95) | 0.033 | 0.58 (0.32– 1.06) | 0.077 |  |  |
| *P* for trend | 0.006 | | 0.008 | | 0.12 | | 0.22 | |  |  |

Model 1: adjusted for age, sex, and race.

Model 2: adjusted for age, sex, race, education, PIR, BMI, smoking, drinking, and total energy.

Model 3: adjusted for all variables in Model 2 plus total PUFA, total fiber, total sodium, total potassium, and total calcium.

Model 4: adjusted for all variables in Model 3 plus hypertension, diabetes, hyperlipidemia, congestive heart failure, coronary heart disease, smoking, drinking, and serum creatinine.

BMI, body mass index; CI, confidence interval; HR, hazard ratio; PIR, ratio of family income to poverty; PUFA, polyunsaturated fatty acids.
